# Forty Years After Chernobyl: Radiocaesium in Wild Edible Mushrooms from North-Eastern Poland and Its Relevance for Dietary Exposure and Food Safety †

**DOI:** 10.3390/toxics13070601

**Published:** 2025-07-17

**Authors:** Iwona Mirończuk-Chodakowska, Jacek Kapała, Karolina Kujawowicz, Monika Sejbuk, Anna Maria Witkowska

**Affiliations:** 1 Department of Food Biotechnology, Medical University of Bialystok, Szpitalna 37, 15-295 Bialystok, Poland; karolina.kujawowicz@sd.umb.edu.pl (K.K.); monika.sejbuk@sd.umb.edu.pl (M.S.); anna.witkowska@umb.edu.pl (A.M.W.); 2 Department of Biophysics, Medical University of Bialystok, Mickiewicza 2A, 15-222 Bialystok, Poland; jacek.kapala@umb.edu.pl

**Keywords:** cesium-137, potassium-40, wild mushrooms, dietary exposure, food, soil

## Abstract

Wild-growing edible mushrooms are known to bioaccumulate radionuclides from their environment, particularly the natural isotope potassium-40 (^40^K) and anthropogenic cesium-137 (^137^Cs). However, region-specific data for commercially relevant species in north-eastern Poland remain limited, despite the cultural and economic importance of mushroom foraging and export. This study aimed to assess the radiological safety of wild mushrooms intended for human consumption, with particular attention to regulatory compliance and potential exposure levels. In this study, 230 mushroom samples representing 19 wild edible species were analyzed using gamma spectrometry, alongside composite soil samples collected from corresponding foraging sites. The activity concentration of ^137^Cs in mushrooms ranged from 0.94 to 159.0 Bq/kg fresh mass (f.m.), and that of ^40^K from 64.4 to 150.2 Bq/kg f.m. None of the samples exceeded the regulatory limit of 1250 Bq/kg f.m. for ^137^Cs. The highest estimated annual effective dose was 2.32 µSv from ^137^Cs and 0.93 µSv from ^40^K, with no exceedance of regulatory limits observed in any sample. A strong positive correlation was observed between ^137^Cs activity in soil and mushroom dry mass (Spearman’s Rho = 0.81, *p* = 0.042), supporting predictable transfer patterns. Additionally, the implications of mushroom drying were assessed considering Council Regulation (Euratom) 2016/52, which mandates radionuclide levels in dried products be evaluated based on their reconstituted form. After such adjustment, even the most contaminated dried samples were found to comply with food safety limits. These findings confirm the radiological safety of wild mushrooms from north-eastern Poland and contribute novel data for a region with limited prior monitoring, in the context of current food safety regulations.

## 1. Introduction

Mushrooms, members of the kingdom Fungi, represent a taxonomically and ecologically diverse group of macrofungi, with fruiting bodies that are widely consumed in many parts of the world [1]. In Poland, the purchase of selected mushroom species is permitted under the Regulation of the Minister of Health [2]. However, there are many more edible mushroom species not included in this regulation that are collected by foragers.

Mushrooms are increasingly recognized as functional foods due to their richness in bioactive compounds. They are natural sources of antioxidants, polyphenols, ergothioneine, essential minerals (such as potassium, selenium, and zinc), as well as biologically active polysaccharides including β-glucans, which contribute to their health-promoting potential [3,4,5,6,7,8]. Beyond their culinary and nutritional value, mushrooms are known for their ability to absorb a wide range of elements from their growing environment. This capacity arises from their extensive mycelial networks, which facilitate efficient uptake and accumulation of both essential bioelements—such as potassium, magnesium, and trace metals—as well as potentially harmful substances, including heavy metals and radionuclides [9,10,11].

Among the contaminants of greatest concern in wild mushrooms and plants are radionuclides, particularly anthropogenic ^137^Cs (cesium-137) [12,13]. The presence of ^137^Cs in terrestrial and marine ecosystems is primarily the result of atmospheric nuclear weapons testing conducted during the mid-20th century and two major nuclear accidents: the Chernobyl disaster in 1986 and the Fukushima Daiichi nuclear accident in 2011 [11,12,13,14,15,16,17,18,19,20,21,22,23]. While the global impact of nuclear testing contributed to the widespread dispersion of radionuclides, the Chernobyl accident had a particularly significant effect on Central, Eastern, South Europe and even Southeast Asia [24,25]. Fallout from this event resulted in the long-term contamination of forest ecosystems, including parts of Poland, where radionuclides were deposited through precipitation and became bound to organic-rich soil layers, facilitating their uptake by fungi and other organisms [26].

More recently, the Fukushima disaster released substantial quantities of radionuclides into the environment, among them ^137^Cs, which has since been detected in various ecological compartments, including wild mushrooms in the affected areas of Japan [27]. These findings have reinforced global concerns about the persistence and mobility of radionuclides in forested ecosystems and the associated risks to human and environmental health [28].

North-eastern Poland is an area of particular interest in this context. It is one of the most forested regions in the country and includes three forest complexes classified as “puszcza”: the Białowieża, Knyszyńska, and Augustów Forests. These extensive and ecologically valuable woodlands are characterized by high biodiversity, minimal human impact, and legally protected areas, making them well-suited for studies on the accumulation of natural and artificial radionuclides in wild mushrooms. Notably, this region was among those affected by radioactive fallout following the Chernobyl nuclear accident in 1986, and forest ecosystems—due to their structure and retention capacity—have continued to serve as long-term reservoirs for radionuclides such as ^137^Cs [29]. Additionally, the area is marked by low levels of industrialization, diverse forest types, and a widespread tradition of wild mushroom foraging. Together, these features provide a relevant context for studying environmental radioactivity and its implications for human dietary exposure. From a scientific perspective, the relatively undisturbed nature of the region enables a clearer assessment of radionuclide distribution and behavior in soils and biota, while minimizing anthropogenic confounding factors.

Naturally occurring ^40^K, an essential nutrient for both fungi and humans, is another radionuclide of interest. Although its radiological significance is generally lower—due to its biological role and relatively stable environmental distribution—it still contributes to the total internal radiation dose received by consumers of wild mushrooms [30,31].

Given the ongoing ecological significance of radionuclide contamination and the role of mushrooms as both a dietary component and environmental indicator, continued surveillance of radiocaesium and potassium-40 levels remains essential. However, north-eastern Poland—despite being one of the most forested and ecologically valuable regions in the country—remains underrepresented in recent radioecological assessments. Integrated analyses of both mushrooms and their corresponding soils are scarce, limiting the understanding of species- and site-specific accumulation patterns.

This study aimed to estimate the potential radiological exposure resulting from the consumption of wild edible mushrooms, based on the determination of ^137^Cs and ^40^K activity concentrations in their fruiting bodies and corresponding soil samples, collected from forested areas of north-eastern Poland.

## 2. Materials and Methods

### 2.1. Research Material

The research material consisted of 19 species of wild-growing edible mushrooms: (1) *Armillaria sp.*; (2) *Boletus edulis* Bull. (3) *Cantharellus cibarius* Fr.; (4) *Cortinarius caperatus* (Pers.) Fr.; (5) *Hydnum repandum* L.; (6) *Imleria badia* (Fr.) Fr.; (7) *Lactarius deliciosus* (L.) Pers.; (8) *Leccinum aurantiacum* (Bull.) Gray; (9) *Macrolepiota procera* (Scop.) Singer; (10) *Russula aeruginea* Lindblad ex Fr.; (11) *Sarcodon imbricatus* (L.) P. Karst.; (12) *Suillus bovinus* (L.) Roussel; (13) *Suillus grevillei* (Klotzsch) Singer; (14) *Suillus luteus* (L.) Roussel; (15) *Suillus variegatus* (Sw.) Richon & Roze; (16) *Tricholoma equestre* (L.) P. Kumm.; (17) *Tricholoma portentosum* (Fr.) Quel.; (18) *Xerocomellus chrysenteron* (Bull.) Šutara; (19) *Xerocomus subtomentosus* (L.) Quél. Of them, *Russula aeruginea*, *Sarcodon imbricatus* and *Tricholoma portentosum* are not permitted for commercial sale under current Polish food safety regulations [2,32]. Although these species are not authorized for commercial sale, they may still be collected by foragers for personal use and are commonly harvested in forested areas.

Mushroom identification was conducted using standard taxonomic keys and field guides and was verified by a certified mushroom classifier. The edible parts of the fruiting bodies (caps and stipes) were cleaned, weighed, homogenized, and lyophilized to a constant dry mass (LyoQuest 55, Telstar, Veslen, The Netherlands). Samples were collected between 2017 and 2021.

### 2.2. Instruments and Equipment

Sample Preparation: Mushroom samples were homogenized using a laboratory grinder (model Raven EMDK003X, Raven, Londz, Poland) and lyophilized to constant dry mass using a freeze dryer (LyoQuest 55, Telstar, The Netherlands).

Gamma Spectrometry: Activity concentrations of radionuclides were measured using a high-purity germanium (HPGe) semiconductor detector (model GX3020-7500SL-RDC-4, Mirion Technologies, Atlanta, GA, USA) with an active volume of 135 cm^3^ (60.5 mm diameter, 47 mm height). The detector featured the following:

- Relative efficiency: 30% (34.8% at 1.33 MeV for ^60^Co, per the manufacturer’s calibration certificate);
- Energy resolution: 1.72 keV at 1.33 MeV (^60^Co) and 0.883 keV at 122 keV (^57^Co);
- Peak-to-Compton ratio: 68.4:1 at 1.33 MeV;
- Energy range: 50 keV—10 MeV;
- Power supply: 3000 V DC;
- Cryostat: Cryo-Cycle Hybrid Cryostat;
- Spectrum analysis: Digital Spectrum Analyzer DSA-1000.

Shielding: The detector was housed in a 50 × 50 × 50 cm shielding chamber composed of a 10 cm-thick low-background lead, internally lined with 1.5 mm copper and 0.5 mm aluminum to absorb secondary X-rays produced by gamma-ray interactions with lead.

Software: Genie-2000 (Canberra, Mirion Technologies, Atlanta, GA, USA)—used for spectrum acquisition and quantitative analysis.

### 2.3. Sampling Locations

**Figure 1:**
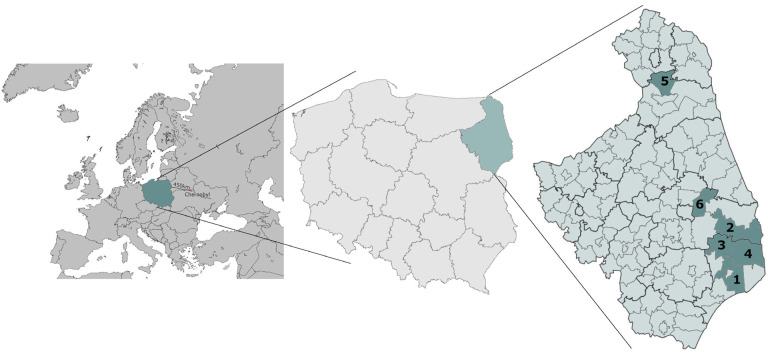
A map of the study area [33]: (1) Hajnówka, (2) Michałowo, (3) Narew, (4) Narewka, (5) Nowinka, (6) Supraśl.

All samples in this study were collected from forested areas within major woodland complexes in north-eastern Poland, rather than from urbanized or residential zones (Figure 1). Although nearby municipalities—Hajnówka, Michałowo, Narew, Narewka, Nowinka, and Supraśl—are used to label the sampling sites, the actual sampling locations are situated in surrounding forests. Specifically, the samples originated from within the Białowieża Forest (Hajnówka, Narew, Narewka), the Knyszyńska Forest (Michałowo, Supraśl), and the Augustów Forest (Nowinka). These areas are known for their extensive, ecologically valuable woodland cover and are representative of undisturbed forest ecosystems. The proximity of collection sites to these municipalities is purely administrative and does not reflect land use or habitat type, as all samples were obtained from natural forest environments. From each location, several to several dozen samples of a given mushroom species were collected, with fresh mass (f.m.) ranging from approximately 500 g to 1 kg. In addition, between 2 and 4 soil samples were collected from each site to provide representative material for the area.

Soil samples were taken from the uppermost mineral layer (0–10 cm) in quantities of approximately 500 g. At each site, five subsamples were collected within a 1 m^2^ area and thoroughly mixed in the field to create a composite sample, ensuring representativeness and homogeneity. Visible impurities such as stones, leaves, branches, insects, and other debris were manually removed. The remaining material was sieved through a plastic mesh (≤2 mm) to obtain a uniform substrate. The prepared, sieved soil was retained for subsequent radiometric analysis.

The aim of the study was to compare the same species of wild forest mushrooms collected from different municipalities. However, despite the focus on relatively common species, not all of them were present at every sampling site. Therefore, only the species naturally occurring in each locality were collected.

Table 1 summarizes the species composition, sample size, and associated moisture content.

### 2.4. Analytical Methods

The ^40^K and ^137^Cs contents of mushroom samples were determined by gamma spectrometry with a high-purity germanium (HPGe) semiconductor detector (model GX3020-7500SL-RDC-4 Canberra, Mirion Technologies, Atlanta GA, USA). The detector was shielded by 10 cm of lead, internally lined with 1.5 mm of pure copper and 0.5 mm of pure aluminum. The technical specifications of the system were as follows: relative efficiency of 34.8% and resolution of 2.0 keV at 1.332 MeV. The ^40^K and ^137^Cs contents were determined using characteristic gamma peaks of 1460.8 keV and 661.7 keV, respectively. Measurement of uncertainty was maintained below 10% for the net peak area. Sample counting times ranged from approximately 90,000 to 250,000 s. Spectra were analyzed using Genie-2000 software (Canberra, Mirion Technologies, Atlanta GA, USA). Each measurement was preceded by a background count of equivalent duration. The detector system was efficiency-calibrated using an MBSS2-type reference source (volume: 450 cm^3^, certificate no. 1035-SE-40133-19, reference date: March 20, 2019), provided by the Czech Metrology Institute. The reliability and comparability of results were verified through participation in intercalibration exercises organized by the Polish National Atomic Energy Agency (PAA). Whenever ^137^Cs activity was measured in surface water, powdered milk, dried vegetables, or sand, the results were submitted and included in the final intercomparison reports. Certified reference materials were also used for quality control: IAEA-375 (soil) and IAEA-373 (mushrooms). Detection limits were calculated using the Genie-2000 system as the Minimum Detectable Activity (MDA), following the definition by Currie [34]. The MDA ranged from 0.3 to 1.5 Bq/kg for ^137^Cs and from 7 to 60 Bq/kg for ^40^K. Radionuclide activity concentrations in mushrooms were calculated on a fresh mass basis, taking into account the individual moisture content of each sample.

### 2.5. Calculation of Transfer Factors

The soil-to-mushroom transfer factor (TF) for a given radionuclide is commonly defined as the ratio of its activity concentration in mushroom fruiting bodies to that in the corresponding soil (both expressed on a dry mass basis):

$$TF=\frac{Am}{\begin{array}{c} As \end{array}}$$

Am = activity concentration of the radionuclide in mushrooms (Bq/kg d.m.);
As = activity concentration of the radionuclide in soil (Bq/kg d.m.) [35].

### 2.6. Effective Dose Estimation

To estimate the effective dose resulting from the ingestion of ^137^Cs and ^40^K, the retention function proposed by the International Commission on Radiological Protection (ICRP) was applied, using the following assumptions:

Radiocaesium and radiopotassium are assumed to be completely absorbed from the gastrointestinal tract; therefore, the absorption coefficient (f_1_) is taken as 1.

The potential radiological health risk to humans is expressed as the effective dose (Eeff), given in millisieverts (mSv) per year. The annual effective dose for an adult resulting from the ingestion of ^137^Cs and ^40^K through mushroom consumption can be estimated based on the following equation:

Eeff = X × Z × dc

where

X—average activity concentration of ^137^Cs or ^40^K in mushrooms (Bq·kg^−1^);
Z—annual mushroom consumption by an adult (kg·year^−1^);
dc—effective dose conversion coefficients for ^137^Cs and ^40^K ingested with food by adults (aged 17 and older), with values of 1.3×10^−8^ Sv·Bq^−1^ and 6.2×10^−9^ Sv·Bq^−1^, respectively, as recommended by the International Commission on Radiological Protection (ICRP) [36,37] and the Regulation of the Council of Ministers of 9 September 2021 [38].

Due to the lack of official data, all analyses regarding wild mushroom consumption are based on available estimates and indirect data. The average annual consumption of wild mushrooms in the Podlaskie Province in the 18–60 age group is 1 kg per person (0.95 kg before rounding). This value was estimated based on the maximum purchase of 580 tons in 2008 and demographic data from the Central Statistical Office. Calculations indicate that with this amount of purchase, each adult in this age group could consume ~1 kg of mushrooms per year. The 18–60 age group realistically reflects the professionally and physically active population that can collect and consume mushrooms [39,40].

To better reflect variation in dietary behavior, two additional scenarios were also considered:

− Consumption of 5 kg/year of fresh mushrooms—representing high-end, regionally elevated consumption.
− Consumption of 1 kg/year of dried mushrooms—representing preserved product intake. In this case, activity concentrations were used on a dry mass basis (d.m.), assuming direct consumption without rehydration, thus representing a conservative upper-bound exposure scenario.
− These three intake levels enable a more nuanced and realistic assessment of potential radiological exposure from the consumption of wild mushrooms in both household and commercial contexts.

### 2.7. Statistical Analysis

The statistical significance level was set at α = 0.05, with a Type II error rate (β) set at 0.20, ensuring 80% power to detect meaningful differences or equivalence, where applicable. Data on mass and the radioactive activity of ^40^K and ^137^Cs isotopes in edible mushrooms samples were presented as medians (Mdn) with the interquartile range (IQR, Q1–Q3), where Q1 and Q3 represent the 25th and 75th percentiles, respectively.

To compare ^40^K and ^137^Cs activity across species and locations, the non-parametric Kruskal–Wallis test was applied, as the data did not meet the assumptions of normality (verified with the Shapiro–Wilk test). When significant differences were identified (*p* < 0.05), or when *p*-values approached the significance threshold (e.g., *p* ≈ 0.05), post hoc Dunn tests with Bonferroni or Holm correction for multiple comparisons were conducted to explore pairwise differences between locations. The ordinal epsilon-squared coefficient (ε^2^ordinal) was calculated to determine the effect size, with the following interpretation: < 0.06 (small effect), 0.06–0.14 (moderate effect), and > 0.14 (large effect). Additionally, the Wilcoxon rank-sum test was used to compare two independent groups (e.g., mycorrhizal vs. saprotrophic species) in cases of non-normal data distribution, providing a robust non-parametric method for assessing differences in radionuclide activity concentrations.

Multivariate analysis of variance (MANOVA) using Pillai’s trace was employed to assess the influence of collection location on the combined dependent variables (activity concentrations of ^40^K and ^137^Cs). MANOVA results were reported as Pillai’s trace, F-value, degrees of freedom, and *p*-value.

Spearman’s rank correlation coefficient (Rho) was used to assess the relationships between ^40^K and ^137^Cs activity concentrations. Correlations were considered statistically significant at *p* < 0.05 and were reported with Rho, *p*-values, and 95% confidence intervals (CI_95_%). Additionally, a robust linear regression model using an M-estimator was applied to assess these relationships, offering a complementary analysis that is resilient to outliers. Correlation results were presented with Rho and *p*-values and visualized using scatter plots that included both standard linear regression and robust fitting lines.

To assess the relationships between radionuclide activity concentrations in mushroom fruiting bodies and corresponding soil samples, Spearman’s rank correlation coefficient was calculated separately for ^137^Cs and ^40^K. The analysis used location-specific median values, and significance was determined at *p* < 0.05. The correlation strength was interpreted as follows: 0–0.3 (weak), 0.3–0.7 (moderate), and >0.7 (strong). Scatter plots with regression lines were included to illustrate these associations.

All statistical analyses were performed using R (version 4.3.3; R Core Team, 2024, Vienna, Austria).

## 3. Results

### 3.1. Radioactivity of Soil Samples

**Figure 2:**
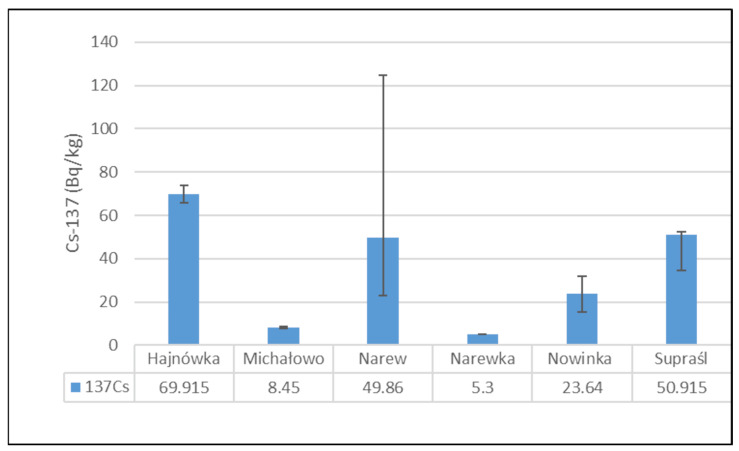
Mean activity concentration of ^137^Cs in soil samples collected from six municipalities in north-eastern Poland, with standard deviations indicated.

Figure 2 presents the mean activity concentrations and standard deviations of ^137^Cs in soil samples collected from six municipalities in north-eastern Poland. The lowest radioactivity was recorded in the Narewka municipality, with an average activity concentration of 5.3 ± 0 Bq/kg. In contrast, the highest level of ^137^Cs radioactivity was observed in soil from the Hajnówka municipality, where the average activity concentration reached 69.92 ± 5.72 Bq/kg. The widest range between minimum and maximum values was noted in samples from the Narew municipality.

**Figure 3:**
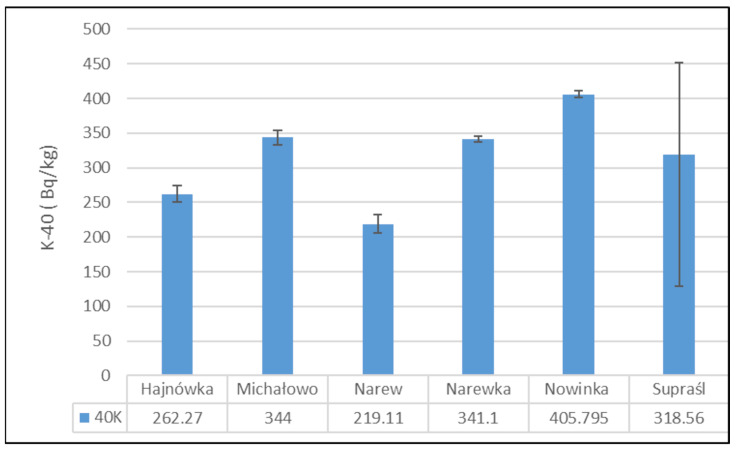
Mean activity concentration of ^40^K in soil samples collected from six municipalities in north-eastern Poland, with standard deviations indicated.

Figure 3 presents the mean activity concentrations and standard deviations of ^40^K in soil samples collected from six municipalities in north-eastern Poland. The lowest ^40^K activity concentration was recorded in the soil from the Narew municipality, with a mean value of 219.11 ± 19.05 Bq/kg. In contrast, the highest activity concentration was observed in the Nowinka municipality, reaching 405.80 ± 6.93 Bq/kg. The widest range between minimum and maximum values was noted in soil samples from the Supraśl municipality.

### 3.2. Radioactivity Concentration of Mushroom Samples

Table 2 presents the median values along with the first (Q1) and third (Q3) quartiles for ^137^Cs and ^40^K activity concentrations in both fresh mass (f.m.) and dry mass (d.m.) of the analyzed mushroom species. The ^137^Cs activity concentration ranged from 8.94 Bq/kg d.m. in *Macrolepiota procera* to 1976.7 Bq/kg d.m. in *Sarcodon imbricatus*. For ^40^K, activity levels ranged from 657.9 Bq/kg d.m. in *Suillus bovinus* to 1787.5 Bq/kg d.m. in *Hydnum repandum* (Table 2). In terms of f.m., the lowest activity of ^137^Cs was observed in *Macrolepiota procera* (0.94 Bq/kg f.m.), and the highest in *Sarcodon imbricatus* (159.0 Bq/kg f.m.). The ^40^K activity concentrations in f.m. ranged from 64.4 Bq/kg in *Suillus bovinus* to 150.2 Bq/kg in *Tricholoma equestre*.

### 3.3. Soil-to-Mushroom Transfer Factors

Transfer factors for ^40^K and ^137^Cs were calculated for each of the 19 mushroom species, with results shown in Table 3. The highest ^40^K accumulation was observed in *Hydnum repandum* (TF = 7.99), while the highest ^137^Cs accumulation was found in *Sarcodon imbricatus* (TF = 41.86).

### 3.4. Mean Effective Doses Calculation

Table 4 presents the estimated effective doses (in µSv/year) resulting from the ingestion of wild mushrooms containing ^137^Cs and ^40^K, assuming three consumption scenarios: 1 kg and 5 kg of fresh mushrooms, and 1 kg of dried mushrooms. The calculations are based on the mean activity concentrations determined for each species and apply ingestion dose conversion coefficients recommended by the ICRP.

For ^137^Cs, the highest effective dose was associated with *Sarcodon imbricatus*, reaching 2.32 µSv for 1 kg of fresh mushrooms and 29.3 µSv for 1 kg of dried product. Other species with notably elevated ^137^Cs-derived doses include *Suillus variegatus* (1.11 µSv/1 kg f.m.) and *Cortinarius caperatus* (1.02 µSv/1 kg f.m.).

For ^40^K, effective doses were generally lower than for ^137^Cs in dried mushrooms but higher in fresh ones, due to the more uniform presence of this natural radionuclide across species. The highest ^40^K-derived dose was observed for *Tricholoma equestre* (0.93 µSv for 1 kg fresh, 9.29 µSv for 1 kg dry), followed by *Macrolepiota procera* and *Tricholoma portentosum.*

Across all evaluated scenarios, none of the calculated combined doses from ^137^Cs and ^40^K exceeded or approached the public exposure limit of 1 mSv/year as defined by the International Commission of Radiological Protection (ICRP) and adopted in EU radiation protection legislation even in the high-consumption or dried-product cases. The highest total dose (^137^Cs + ^40^K) was 35.89 µSv/year for *Sarcodon imbricatus* (1 kg dried).

### 3.5. Assessment of ^137^Cs Activity Concentration in Dry Mass Across Species

**Figure 4:**
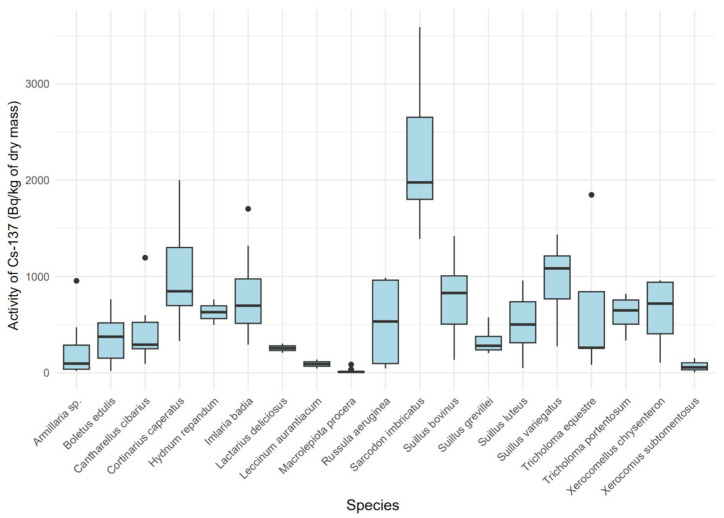
Variability in ^137^Cs activity [Bq/kg dry mass] by mushroom species.

The activity concentration of ^137^Cs in dry mass exhibited significant variability among species (Figure 4) (Kruskal–Wallis, χ^2^ = 87.42 (18), *p* < 0.001), indicating a substantial influence of the mushroom’s ecological type on the accumulation of this isotope. Mycorrhizal species, such as *Sarcodon imbricatus* (median: 1957.6 Bq/kg dry mass, IQR: 1414.7–3318.6) and *Cortinarius caperatus* (median: 811.23 Bq/kg dry mass, IQR: 329.05–1858.03), showed higher ^137^Cs concentrations compared to saprotrophic species, such as *Macrolepiota procera* (median: 5.87 Bq/kg dry mass, IQR: 2.79–15.62). A boxplot (Figure 4) revealed distinct groups: species with high ^137^Cs accumulation (*Sarcodon imbricatus*, *Cortinarius caperatus*, *Imlaria badia*) and those with low accumulation (*Macrolepiota procera*, *Xerocomus subtomentosus*).

### 3.6. Assessment of ^40^K Activity in Dry Mass Across Species

**Figure 5:**
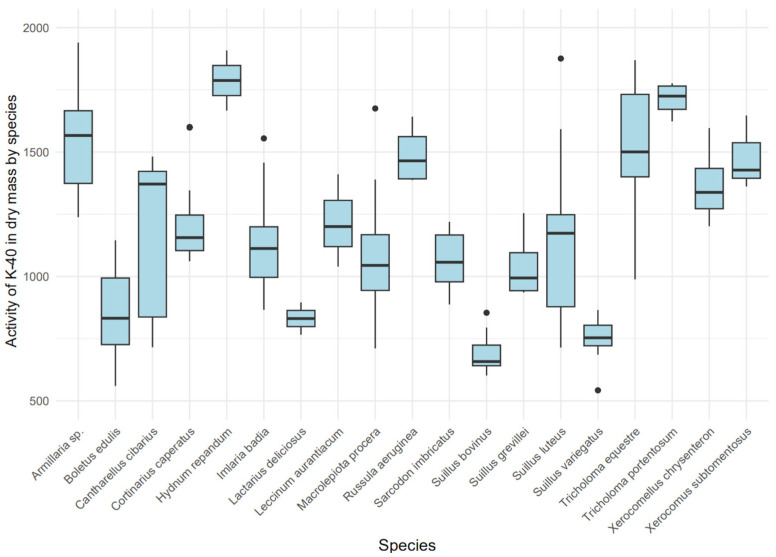
Variability in ^40^K activity [Bq/kg dry mass] among mushroom species.

The activity concentration of ^40^K in the dry mass of various mushroom species exhibited significant variability (Kruskal–Wallis, χ^2^ = 92.14 (18), *p* < 0.001) (Figure 5), indicating a strong influence of ecological type on ^40^K accumulation. Mycorrhizal species, such as *Hydnum repandum* (median: 1787.45 Bq/kg dry mass, IQR: 1726.97–1847.93) and *Tricholoma portentosum* (median: 1724.70 Bq/kg dry mass, IQR: 1671.73–1765.25), demonstrated higher ^40^K concentrations compared to saprotrophic species like *Macrolepiota procera* (median: 1044.61 Bq/kg dry mass, IQR: 943.77–1167.82).

A boxplot analysis in Figure 5 revealed distinct groups: species with high ^40^K accumulation, including *Hydnum repandum*, *Tricholoma portentosum*, and *Armillaria* sp. (median: 1567.04 Bq/kg dry mass, IQR: 1382.60–1696.30), and species with lower accumulation, such as *Suillus bovinus* (median: 657.90 Bq/kg dry mass, IQR: 641.35–724.12) and *Suillus variegatus* (median: 753.50 Bq/kg dry mass, IQR: 721.55–803.92). Notably, *Armillaria* sp. exhibited exceptionally high ^40^K levels, potentially due to its parasitic feeding mode, while *Macrolepiota procera* showed greater variability but lower maximum values compared to most mycorrhizal species.

### 3.7. Assessment of Relationships Between ^40^K and ^137^Cs Activity Concentrations

The analysis was conducted using data on the activity concentrations of ^40^K and ^137^Cs in both fresh and dry mass of edible mushrooms. Relationships between these radionuclides were assessed using scatter plots with linear regression lines and 95% confidence intervals. Spearman’s rank correlation coefficient (Rho) and the corresponding *p*-values were calculated to evaluate the strength and significance of the associations.

#### 3.7.1. Assessment of the Relationship Between ^40^K and ^137^Cs Activity in Mushroom d.m.

**Figure 6:**
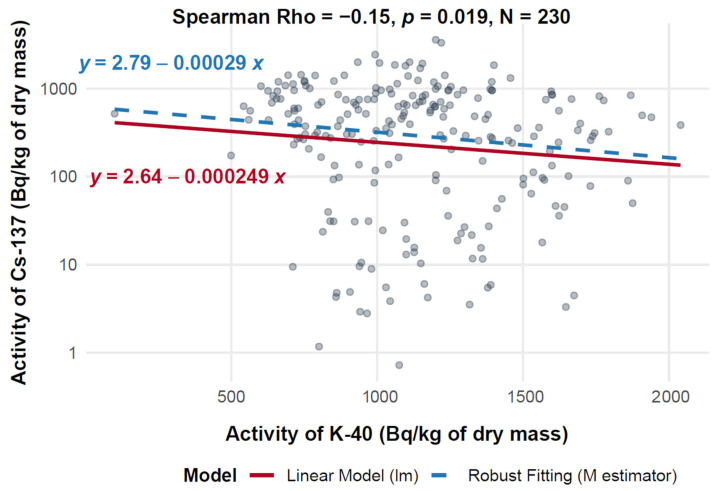
Relationship between ^40^K and ^137^Cs activity in d.m. of mushrooms (logarithmic scale).

Figure 6 presents a scatter plot illustrating the relationship between ^40^K activity (on the *X*-axis, expressed in Bq/kg of dry mass) and ^137^Cs activity (on the *Y*-axis, also in Bq/kg of dry mass) across a dataset of 230 measurements. The analysis includes both ordinary least squares (OLS) linear regression and robust regression, the latter using an M-estimator to limit the influence of outliers. The Spearman correlation coefficient is −0.15 (*p* = 0.019), indicating a statistically significant but weak negative correlation between the two radionuclides.

The dataset shows considerable variability, especially in the ^40^K activity range of 500–1500 Bq/kg, where ^137^Cs activity ranges from 1 to 1000 Bq/kg. At ^40^K levels exceeding 1500 Bq/kg, ^137^Cs activity remains widely scattered, with values ranging from ~1 to over 3000 Bq/kg. The robust regression line, which mitigates the effect of extreme values, shows a slightly shallower slope (−0.000249) compared to the standard linear regression (−0.00029), indicating that outliers had a minor effect on the overall trend. This alignment across methods reinforces the robustness of the observed weak negative association.

#### 3.7.2. Assessment of the Relationship Between ^40^K and ^137^Cs Activity in f.m.

**Figure 7:**
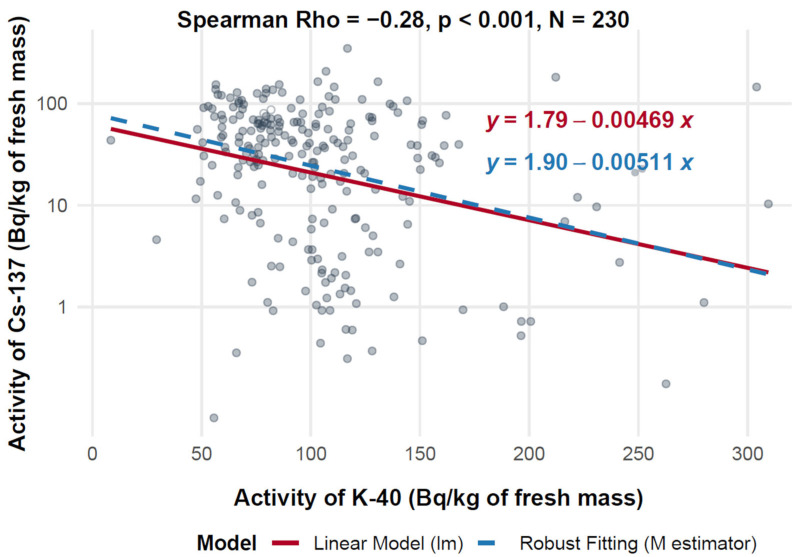
Relationship between ^40^K and ^137^Cs activity in f.m. of mushrooms (logarithmic scale).

Figure 7 presents a scatter plot illustrating the relationship between ^40^K activity (*X*-axis, expressed in Bq/kg of fresh mass) and ^137^Cs activity (*Y*-axis, also expressed in Bq/kg of fresh mass) across a range of 230 measurements. The analysis reveals a statistically significant, though weak, negative correlation, with a Spearman correlation coefficient of −0.28 (*p* < 0.001). This indicates a consistent inverse association between ^40^K and ^137^Cs activity concentrations, observed across diverse geographic locations and mushroom species.

The linear regression model yields the equation log_10_(^137^Cs) = 1.79 − 0.00469 × ^40^K, while the robust regression model provides log_10_(^137^Cs) = 1.90 − 0.00511 × ^40^K. The small difference in slope values (−0.00469 vs. −0.00511) supports the robustness of the observed trend. Across the dataset, ^40^K activity concentrations ranged from 0 to 300 Bq/kg, and ^137^Cs ranged from 1 to 100 Bq/kg.

### 3.8. Assessment of the Effect of Collection Location on ^40^K and ^137^Cs Activity

**Figure 8:**
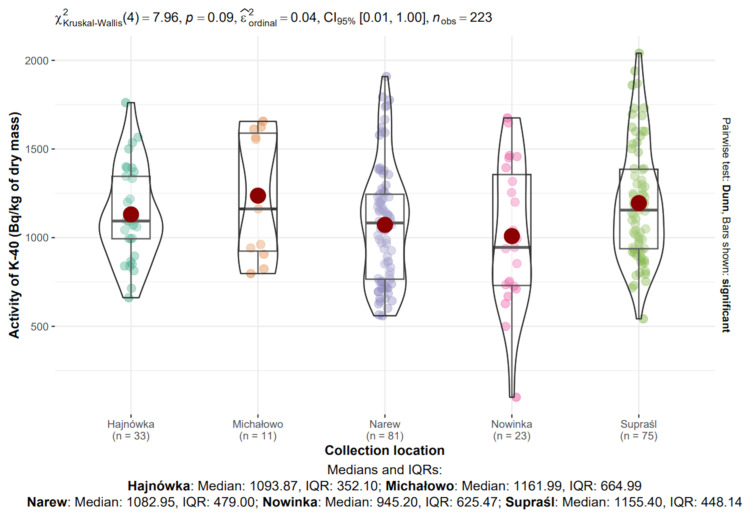
Analysis of ^40^K activity in dry mass depending on the collection location.

**Figure 9:**
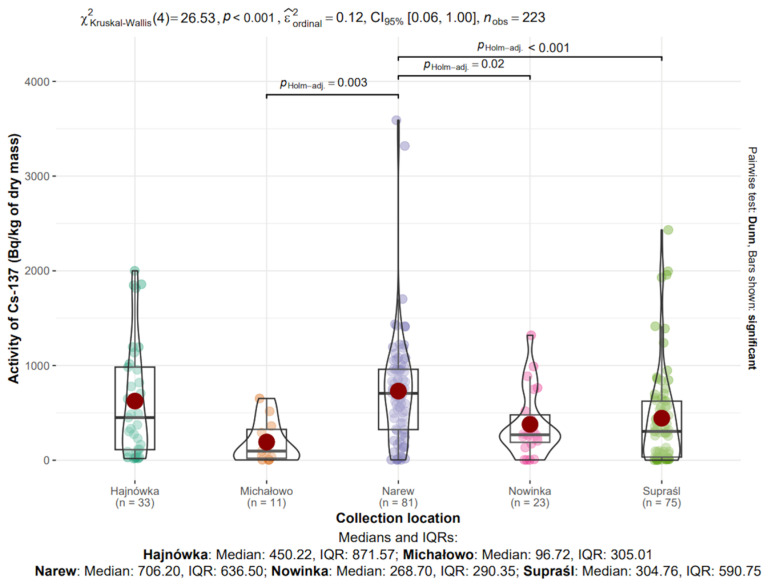
Analysis of ^137^Cs activity in dry mass depending on collection location.

The influence of collection location on the radioactive activity of ^40^K and ^137^Cs isotopes in edible mushroom samples collected between 2017 and 2021 in north-eastern Poland was analyzed. Samples from five locations (the sixth location, Narewka, was excluded from the analysis due to the small sample size, n = 6): Hajnówka (n = 33), Michałowo (n = 11), Narew (n = 81), Nowinka (n = 23), and Supraśl (n = 75), totaling 223 observations. Data distributions are visualized in Figure 8 and Figure 9.

A multivariate analysis of variance (MANOVA) revealed a significant effect of location on the combined activity of ^40^K and ^137^Cs (Pillai = 0.389, F(12, 588) = 3.614, *p* < 0.001), indicating variation in radioactive activity depending on the collection site.

#### 3.8.1. ^40^K in Mushrooms from Different Locations

The median activity concentrations of ^40^K in dry mass across the sampling locations were as follows: Hajnówka—1003.87 Bq/kg (IQR: 352.10), Michałowo—1161.99 Bq/kg (IQR: 664.99), Narew—1082.95 Bq/kg (IQR: 479.00), Nowinka—945.20 Bq/kg (IQR: 625.47), and Supraśl—1155.40 Bq/kg (IQR: 448.14) (Figure 9).

The highest median ^40^K concentration was observed in Michałowo, while the lowest was recorded in Nowinka. The greatest variability, as indicated by the interquartile range, was also noted in Michałowo, possibly reflecting sample heterogeneity within this site.

The Kruskal–Wallis test did not reveal statistically significant differences in ^40^K activity concentrations between locations (χ^2^(4) = 7.96, *p* = 0.09, ε^2^_ordinal_ = 0.04).

#### 3.8.2. ^137^Cs in Mushrooms from Different Locations

The median activity concentrations of ^137^Cs in dry mass were as follows: Hajnówka—450.22 Bq/kg (IQR: 871.57), Michałowo—96.72 Bq/kg (IQR: 305.01), Narew—706.20 Bq/kg (IQR: 636.50), Nowinka—268.70 Bq/kg (IQR: 290.35), and Supraśl—304.76 Bq/kg (IQR: 590.75) (Figure 9).

The highest median value was observed in Narew, whereas the lowest was recorded in Michałowo. The greatest variability, as indicated by the interquartile range, was found in Hajnówka, suggesting considerable heterogeneity in the sampled material from that location.

The Kruskal–Wallis test confirmed statistically significant differences in ^137^Cs activity concentrations among the studied locations (χ^2^(4) = 26.53, *p* < 0.001, ε^2^_ordinal_ = 0.12).

### 3.9. Difference Analysis of the Activity Concentrations of ^40^K and ^137^Cs in Soil Across Different Regions

**Figure 10:**
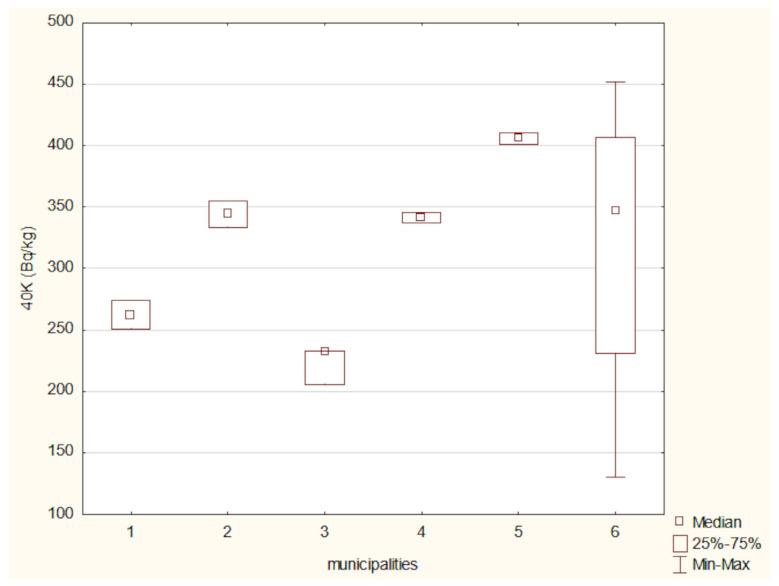
^40^ K activity in soils from the studied locations: (1) Hajnówka, (2) Michałowo, (3) Narew, (4) Narewka, (5) Nowinka, (6) Supraśl.

**Figure 11:**
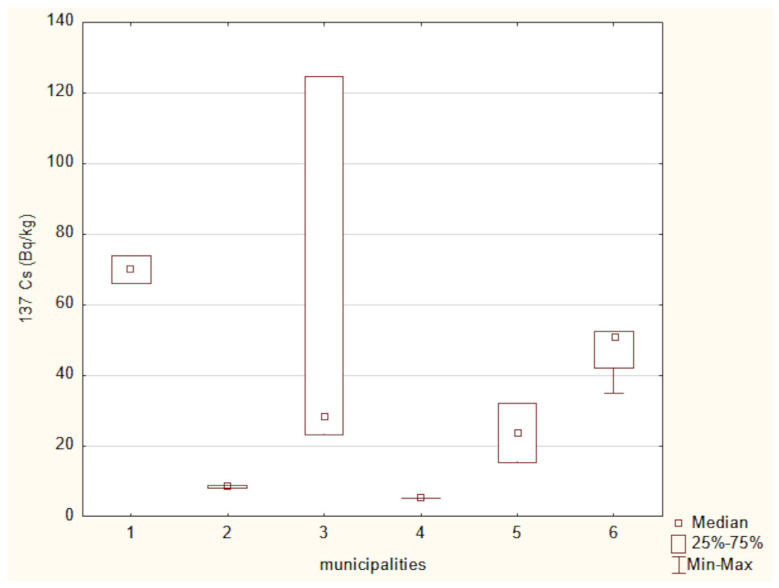
^137^Cs activity in soils from the studied locations: (1) Hajnówka, (2) Michałowo, (3) Narew, (4) Narewka, (5) Nowinka, (6) Supraśl.

The activity concentrations of ^40^K and ^137^Cs in the analyzed soil samples were compared using the Kruskal–Wallis test (Figure 10 and Figure 11). A Kruskal–Wallis test was applied to assess differences in ^40^ K concentrations among the six locations. The result was not statistically significant (χ^2^(5) = 7.96, *p* = 0.158), suggesting that the observed differences may not be robust enough to confirm regional variation at the α = 0.05 level.

A Kruskal–Wallis test for ^137^Cs concentrations across six locations approached statistical significance (χ^2^(5) = 11.04, *p* = 0.051), suggesting potential differences in soil contamination. However, post hoc multiple comparisons using Dunn’s test with Bonferroni correction did not reveal any statistically significant pairwise differences, suggesting that while regional variability in ^137^Cs exists, the differences between individual municipalities may not be strong enough to be statistically distinguishable after correction for multiple testing.

### 3.10. Correlation of ^137^Cs and ^40^K Activity in Soil and Mushroom Dry Mass

**Figure 12:**
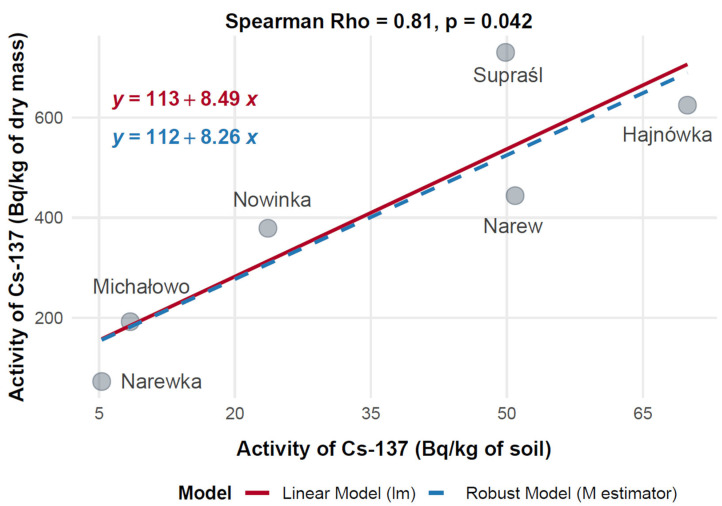
Correlation between ^137^Cs activity concentrations in soil and mushrooms (dry mass) collected from different locations in north-eastern Poland.

To evaluate the relationship between soil contamination and radionuclide uptake in mushrooms, the mean activity concentrations of ^137^Cs in mushroom dry mass (Bq/kg) were compared with corresponding levels in soil (Bq/kg) across six municipalities in Poland: Hajnówka, Michałowo, Narew, Narewka, Nowinka, and Supraśl. Spearman’s rank-order correlation was applied to assess the monotonic relationship between ^137^Cs activity concentrations in soil and mushroom dry mass, with results visualized in Figure 12.

The data showed substantial variation in ^137^Cs activity across the municipalities. Narew exhibited the highest mushroom ^137^Cs activity at 730.14 Bq/kg dry mass, corresponding to a soil activity of 49.86 Bq/kg. Hajnówka followed with 624.74 Bq/kg in mushrooms and 69.92 Bq/kg in soil, indicating elevated contamination levels. Supraśl showed moderate mushroom activity (443.99 Bq/kg) with a soil activity of 50.91 Bq/kg. In contrast, Nowinka and Michałowo recorded lower mushroom activities—378.85 Bq/kg and 192.30 Bq/kg—associated with soil levels of 23.64 Bq/kg and 8.45 Bq/kg, respectively. Narewka presented the lowest values, with 72.80 Bq/kg in mushrooms and 5.30 Bq/kg in soil.

Spearman’s rank correlation analysis revealed a strong positive relationship between ^137^Cs activity in soil and mushrooms, with a correlation coefficient of Rho = 0.81 (*p* = 0.042), indicating that higher levels of ^137^Cs in soil are associated with increased accumulation in mushrooms. Both linear and robust regression models yielded consistent results, confirming a stable positive trend across data points.

This significant correlation supports the use of mushrooms as potential bioindicators of environmental radiocaesium contamination. However, the observed variability between municipalities suggests that local environmental factors—such as soil characteristics, species-specific uptake capacity, and microhabitat differences—may influence radionuclide transfer dynamics.

**Figure 13:**
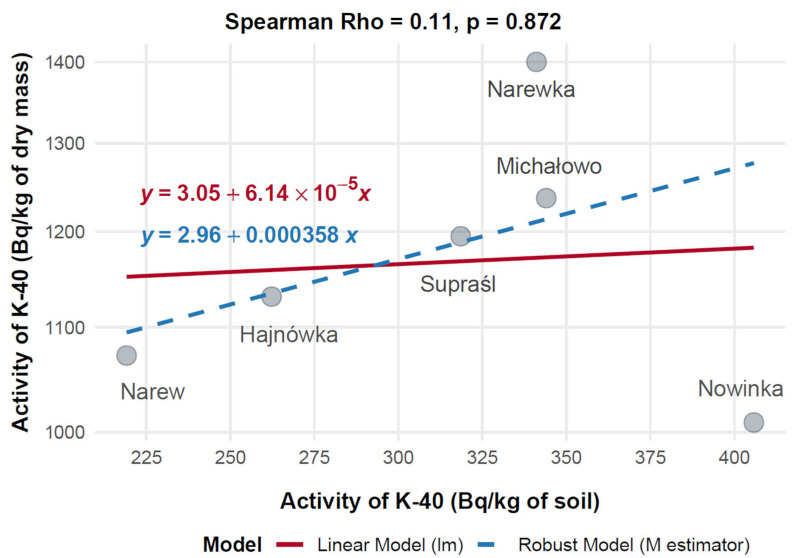
Correlation between ^40^K activity concentrations in soil and mushrooms (dry mass) collected from different locations in north-eastern Poland.

Mean activity concentrations of ^40^K (Bq/kg) in mushroom dry mass and corresponding soil samples were compared across six Polish municipalities—Hajnówka, Michałowo, Narew, Narewka, Nowinka, and Supraśl—to evaluate their relationship (Figure 13). The highest ^40^K activity in mushrooms was observed in Narewka (1400.00 Bq/kg) with a soil activity of 341.10 Bq/kg, followed by Michałowo (1237.03 Bq/kg, 344.00 Bq/kg) and Supraśl (1194.90 Bq/kg, 318.56 Bq/kg). In contrast, Nowinka exhibited the highest soil activity (405.80 Bq/kg) but a lower mushroom activity (1008.91 Bq/kg), while Narew showed 1072.20 Bq/kg in mushrooms despite having the lowest soil activity (219.11 Bq/kg).

Spearman’s rank correlation revealed a very weak, non-significant positive association between soil and mushroom ^40^K activity concentrations (Rho = 0.11, *p* = 0.872, n = 6). A 20% Winsorized correlation analysis produced a moderate, though still non-significant, positive correlation (Rho = 0.52, 95% CI: –0.50 to 0.94, *p* = 0.288), suggesting that high mushroom activity in Narewka may act as an outlier obscuring underlying trends.

Figure 12 illustrates divergence between standard and robust regression models, with the robust model aligning more closely with the Winsorized correlation and providing a more stable estimate. Overall, these findings suggest that ^40^K uptake in mushrooms is not strongly dependent on soil concentrations, implying that local physiological or environmental factors may play a more prominent role in modulating accumulation.

### 3.11. Assessment of ^40^K Activity Concentration in Dry Mass Across Nutritional Strategies

Mycorrhizal species—forming symbiotic associations with plant roots—accounted for 169 samples, while saprotrophic species, which obtain nutrients from decomposing organic matter, comprised 61 samples (see Table A1 in the Appendix A). Mycorrhizal mushrooms exhibited a median ^40^K activity concentration of 1050.90 Bq/kg (IQR: 813.80–1243.54 Bq/kg), which was notably lower than the median of 1324.39 Bq/kg (IQR: 1044.61–1573.23 Bq/kg) observed in saprotrophic species. This difference suggests that mycorrhizal fungi accumulate lower levels of ^40^K, potentially due to distinct nutrient acquisition strategies and physiological regulation mechanisms. While saprotrophic species may rely more directly on potassium released from decaying organic matter, mycorrhizal fungi likely maintain tighter homeostatic control of potassium uptake through regulated exchange with host plants. These findings highlight the influence of ecological and physiological factors on radionuclide accumulation, rather than direct effects of mycelial structure on ^40^K retention [26,31].

### 3.12. Assessment of ^137^Cs Activity Concentration in Dry Mass Across Nutritional Strategies

Mycorrhizal species, which form symbiotic associations with plant roots, exhibited a median ^137^Cs activity concentration of 623.90 Bq/kg (IQR: 303.10–947.77 Bq/kg, n = 169), markedly higher than the median of 24.50 Bq/kg (IQR: 8.94–91.55 Bq/kg, n = 61) observed in saprotrophic species, which derive nutrients from decomposing organic matter. This substantial difference underscores the enhanced accumulation of ^137^Cs in mycorrhizal fungi, likely attributable to their expansive mycelial networks and interactions with organic-rich forest soils known to retain radiocaesium. In contrast, saprotrophic species exhibit more limited uptake capacity due to their dependence on localized organic substrates [18].

These findings align with the distinct nutrient acquisition strategies of the two ecological groups and reinforce the role of mycorrhizal symbiosis in facilitating elevated radionuclide uptake. This occurs despite the essential metabolic role of potassium, which is subject to tighter physiological regulation than cesium [31].

## 4. Discussion

This study focused on cesium-137 (^137^Cs) and potassium-40 (^40^K), two radionuclides of environmental relevance that are frequently detected in soils, mushrooms, and both terrestrial and marine biota [13,18,22,23,24,41]. ^137^Cs is a long-lived artificial radionuclide produced during nuclear fission, with a half-life of approximately 30 years. It was released in substantial quantities during nuclear accidents, most notably the Chernobyl disaster, and remains one of the primary contributors to residual radioactivity in terrestrial ecosystems in Poland. It is also known for its high mobility in the environment and its tendency to be absorbed and accumulated by fungi, making it an ideal tracer for contamination studies and transfer analysis. In contrast, ^40^K is a naturally occurring radionuclide, present ubiquitously in soils and biological tissues due to the essential role of potassium in cellular metabolism. Although it contributes to background radiation, its inclusion in this study provides a reference for natural radioactivity and enables comparison between anthropogenic and natural radionuclide activity in mushrooms and soils. The combined analysis of ^137^Cs and ^40^K enables a more comprehensive evaluation of anthropogenic contamination and naturally occurring radioisotopes, thereby facilitating a clearer assessment of radiological exposure and its ecological implications.

This study investigated the activity levels of ^137^Cs and ^40^K in 19 mushroom species collected from six municipalities in north-eastern Poland, along with composite soil samples representing each area. Soil samples were collected to provide a comprehensive assessment of environmental radionuclide contamination and its potential transfer to biota. The analysis of soil is essential for establishing baseline levels of artificial and natural radionuclides.

### 4.1. Activity of the Soil Samples

The highest ^137^Cs activity was recorded in soil samples collected from the Hajnówka municipality. In contrast, the widest range of activity for this radionuclide was observed in soil samples from the Narew municipality. These differences in ^137^Cs activity among the examined soil samples are most likely attributable to variations in geological soil characteristics, as well as the complex pathways of radioactive air mass transport and localized rainfall patterns following the Chernobyl accident [42]. According to Chibowski et al. [12] ^137^Cs activity in eastern Poland ranges from 1.3 to 124.7 Bq/kg. The ^137^Cs activity levels observed in the present study fall within this reported range.

The activity concentration of potassium-40 (^40^K) in the analyzed soil samples exhibited relatively modest variation between the studied locations. The highest ^40^K activity was recorded in soils from the Nowinka municipality, while the widest range of activity was observed in samples from Supraśl. The elevated ^40^K activity in Nowinka may be associated with the area’s geological background and ecological characteristics, particularly its proximity to Puszcza Augustowska—one of Poland’s largest and least-disturbed forest complexes. The forest’s podzolic and brown soils, developed from glacial sands and loams, often contain varying levels of potassium-bearing minerals, which can contribute to naturally higher levels of ^40^K. The presence of diverse coniferous and mixed forest vegetation may also influence potassium cycling through litterfall and organic matter decomposition, further enriching surface soils with potassium.

Globally, the average ^40^K activity in soil is estimated at approximately 400 Bq/kg [43], whereas measurements specific to eastern Poland range from 132 to 729 Bq/kg [12]. The ^40^K levels obtained in this study fall within this regional range, aligning with historical data from the 1990s, which suggests a relatively stable and consistent presence of this naturally occurring radionuclide in the region’s soils [12].

### 4.2. ^137^Cs in the Analyzed Mushroom and Soil Samples

The presence of radiocaesium (^137^Cs) in the natural environment is of particular concern due to its long physical half-life, high mobility within food chains, and significant bioavailability [24]. Its uptake by organisms is further facilitated by specific soil characteristics, including low pH, low clay content, and high organic carbon content [13]. These properties can enhance the bioavailability of ^137^Cs and promote its accumulation in fungi, making mushrooms effective bioindicators of radioactive contamination [31]. Mushrooms are among the primary contributors to internal radiation exposure from radiocaesium in populations living in forested areas contaminated by nuclear accidents, particularly in regions where wild mushrooms constitute a regular part of the diet [44]. In contrast, ^210^Po from seafood may contribute more significantly to internal dose in coastal populations, especially where marine products are a dietary staple. These differences highlight the importance of regional dietary habits when assessing radiological risk [45]. The ^137^Cs activity observed in the analyzed mushroom species exhibited substantial variation between taxa. Previous studies have demonstrated that mycorrhizal fungi tend to accumulate significantly higher levels of radiocaesium compared to saprotrophic species [46]. This difference is likely related to the distinct ecophysiological traits and nutrient acquisition mechanisms characteristic of these fungal groups [46]. In the present study, the lowest levels of ^137^Cs activity were recorded in saprotrophic mushroom species, particularly *Macrolepiota procera*. Similarly, low radiocaesium concentrations in *Macrolepiota procera* fruiting bodies have also been reported in previous studies, supporting the observation that saprotrophs generally exhibit lower radiocaesium accumulation compared to mycorrhizal species [47]. Relatively low ^137^Cs activity, compared to that found in mycorrhizal fungi, was also observed in *Armillaria* sp., which is consistent with findings reported by other authors [48]. Among the mycorrhizal species, the lowest ^137^Cs activity was observed in *Xerocomus subtomentosus* and *Leccinum aurantiacum*. In the present study, the activity of ^137^Cs in *L. aurantiacum* was found to be nearly three times lower than that reported in the same species collected in Ukraine, approximately 30 years after the Chernobyl nuclear power plant accident [45]. *Boletus edulis* is one of the most popular mycorrhizal mushroom species among foragers. In the present study, the ^137^Cs activity in this species was found to be an order of magnitude lower compared to specimens collected in Poland between 1999 and 2007 [49], and two orders of magnitude higher than that reported in *B. edulis* collected in China in 2016 [50]. The observed differences in ^137^Cs content between mushrooms from Poland and China may be attributed to the varying bioavailability of ^137^Cs in different environments. This bioavailability is influenced by factors such as soil pH and organic matter content, with more acidic soils and higher organic matter levels facilitating the uptake of ^137^Cs by plants and fungi [13,51]. ^137^Cs content in *Boletus edulis* from this study was also generally lower than the levels reported for the same species collected in Poland between 1995 and 2006 [18]. These findings suggest that the primary source of radiocaesium detected in Polish mushrooms is the fallout from the Chernobyl accident, and that over time, the ^137^Cs content in soils is gradually declining, which in turn reduces the bioavailability of this radionuclide to fungi.

As demonstrated in previous studies, mycorrhizal (symbiotic) fungi exhibit a greater tendency to accumulate ^137^Cs, partly due to the deeper location of their mycelium and their direct interaction with the roots of trees and shrubs [46]. Additionally, mushrooms collected from forest organic soils tend to show higher levels of cesium compared to those growing on mineral soils in meadow environments. Furthermore, mushrooms from coniferous forests generally contain higher ^137^Cs concentrations than those from deciduous forests, a difference attributed to the lower pH of soils under coniferous stands [52]. Other important factors influencing radiocaesium levels in mushrooms include the depth of mycelial growth and the ecophysiological characteristics of the fungal species. Indeed, different species of edible fungi vary significantly in their ability to accumulate radionuclides [31]. Moreover, some studies have shown that young fruiting bodies may contain substantially higher concentrations of ^137^Cs compared to fully mature ones [53].

In the present study, the highest ^137^Cs concentration in dry matter was recorded in *Sarcodon imbricatus*. Slightly lower, yet still elevated, levels of ^137^Cs were found in *Cortinarius caperatus*, *Suillus variegatus*, *Suillus bovinus*, and *Imlaria badia*. Similarly high levels of radiocaesium in *C. caperatus* were previously reported in Polish studies by Falandysz et al. [54]. In the case of *I. badia*, our findings are consistent with previous research indicating the species’ notable capacity to accumulate ^137^Cs. Despite the relatively high ^137^Cs activity in *I. badia* observed in this study compared to other species, the levels were approximately eight times lower than those reported in earlier Polish studies by Malinowska et al. in 2006 [31]. This discrepancy may be attributed to the time gap between sample collections, as the mushrooms analyzed in the present study were collected approximately 15 years later, allowing for a natural decline in soil cesium levels due to radioactive decay and environmental redistribution [55,56]. The highest transfer factor was found for *Sarcodon imbricatus*, followed by *Cortinarius caperatus* and *Imlaria badia*, respectively. No previous studies were found regarding ^137^Cs accumulation in *Sarcodon imbricatus* in Poland.

The ^137^Cs activity in mushroom fruiting bodies examined in this study was generally higher than in Mediterranean mussels and lower than in macroalgae from the same region, and exceeded the levels reported for annual and perennial plants studied by Abbasi et al. These comparisons should be interpreted with caution due to differences in ecosystem type and uptake mechanisms, but they illustrate the notable accumulation potential of wild mushrooms in forest environments [13,23].

Differences in ^137^Cs activity between the sampling locations confirm that the place of collection affects the level of radiocaesium in mushrooms. Higher activity levels were consistently recorded in samples from Narew and Hajnówka, while lower values were found in Michałowo and Nowinka. These findings demonstrate that spatial variation contributes to differences in contamination levels, regardless of species.

The high mobility and bioavailability of ^137^Cs in forest ecosystems contribute to pronounced interspecies variability in mushroom uptake, primarily driven by spatial heterogeneity in soil contamination.

Soil ^137^Cs activity concentrations varied considerably across sampling locations, ranging from 5.30 Bq/kg in Narewka to 69.92 Bq/kg in Hajnówka. These disparities reflect uneven deposition from the Chernobyl accident, shaped by local precipitation patterns and soil organic matter content [43]. Corresponding ^137^Cs activity in mushrooms mirrored this variability, with the highest median values recorded in Narew (730.14 Bq/kg) and Hajnówka (624.74 Bq/kg), aligning with soil levels of 49.86 Bq/kg and 69.92 Bq/kg, respectively. In contrast, lower mushroom activities were observed in Narewka (72.80 Bq/kg) and Michałowo (192.30 Bq/kg), which also exhibited lower soil activities of 5.30 Bq/kg and 8.45 Bq/kg. Intermediate values were found in Nowinka and Supraśl, with mushroom activities of 378.85 Bq/kg and 443.99 Bq/kg corresponding to soil concentrations of 23.64 Bq/kg and 50.91 Bq/kg, respectively.

Spearman’s rank correlation revealed a strong, statistically significant positive relationship between soil and mushroom ^137^Cs activity (Rho = 0.82, *p* = 0.042, n = 6), confirming that spatial heterogeneity in soil contamination is a major determinant of fungal uptake. Power analysis indicated high statistical power (0.75 for Rho = 0.82, α = 0.05), further validating the robustness of this association.

Interspecies variability reinforced these spatial trends, especially among mycorrhizal species adapted to organic-rich forest soils that preferentially retain radiocaesium [46]. For instance, *Sarcodon imbricatus* displayed a median ^137^Cs activity of 1957.6 Bq/kg, while *Cortinarius caperatus* exhibited 811.23 Bq/kg, both markedly higher than saprotrophic species. These differences underscore the asymmetrical distribution of ^137^Cs across species and sites, attributable to heterogeneous fallout from the Chernobyl disaster [42].

Taken together, these findings emphasize the utility of mycorrhizal mushrooms as sensitive bioindicators of environmental ^137^Cs contamination. Their uptake patterns reflect both ecological specialization and local contamination gradients, supporting their use in long-term monitoring of radiological persistence in post-Chernobyl forested landscapes.

### 4.3. ^40^K in the Analyzed Mushroom and Soil Samples

^40^K is a natural component of the total potassium content, which is an essential nutrient and one of the most abundant elements in mushroom fruiting bodies—both mycorrhizal and saprotrophic species. The reported activity levels of this radionuclide in mushrooms typically range from 800 to 1500 Bq/kg dry mass (d.m.) [57]. ^40^K is one of the most commonly studied natural radionuclides in food. Unlike ^137^Cs, no significant differences have been observed in relation to the nutritional mode of fungi. Both mycorrhizal and saprotrophic mushrooms exhibit similar levels of ^40^K [58]. In the present study, the activity concentrations of ^40^K in the analyzed mushroom species ranged from 657.9 Bq/kg d.m. in *Suillus bovinus* to 1787.5 Bq/kg d.m. in *Hydnum repandum*. Relatively low levels of this radionuclide were also observed in *Suillus variegatus* and *Boletus edulis*. The concentrations of ^40^K in these species are consistent with values reported in previous studies [18]. *B. edulis* collected from diverse geographical locations has been shown to exhibit comparable activity levels of ^40^K, which may reflect not only similar concentrations of this radionuclide in soil but also the species’ specific physiological requirements [18]. The highest concentrations of ^40^K in this study were recorded in *Hydnum repandum* and *Tricholoma portentosum*, corroborating findings from earlier research, in which these species also demonstrated elevated levels of potassium-40 [57]. *Armillaria* sp. was also characterized by a high ^40^K content. The combination of low ^137^Cs and high ^40^K levels in *Armillaria* sp. fruiting bodies may be attributed to their growth on tree trunks, with the host trees accessing nutrients through deep root systems in soil layers where ^137^Cs availability is limited [59]. Furthermore, when both cesium and potassium are present, plants preferentially absorb potassium, as cesium is not an essential element for plant metabolism [60]. Potassium, by contrast, is an essential nutrient whose absorption by plants and fungi is self-regulated according to their physiological requirements [57]. A single *Armillaria* sp. organism can extend its mycelium and rhizomorphs over an area of up to 150,000 m^2^ of forest. The presence of large amounts of ^40^K in its fruiting bodies may be attributed to the role of rhizomorphs as highly efficient structures for the uptake of water and minerals from the soil [61]. The consistency in ^40^K activity across different locations for the same species suggests that species-specific physiological factors are the dominant drivers of potassium accumulation. Accordingly, several studies have failed to identify statistically significant correlations between ^40^K levels in soil and corresponding mushroom samples, further supporting the hypothesis that potassium uptake is under tight physiological regulation by the fungal organism [57]. Moreover, previous research has indicated that the maturity of the fruiting body—often correlated with its size—can influence the accumulation of ^40^K, with more mature specimens generally exhibiting higher activity levels [53]. In general, ^40^K activity remains relatively constant and characteristic for a given species, and does not correlate with the activity of anthropogenic radionuclides such as ^137^Cs [62]. This stability is attributed to the essential role of potassium as a macroelement in fungal metabolism and its homeostatic regulation within the organism [31]. However, in our own studies, a weak, negative correlation was found between the content of ^40^K and ^137^Cs. The negative correlation may be explained by the fact that plants and mushrooms absorb cesium via potassium transporters, despite cesium having no known beneficial function in biological systems. When potassium levels are elevated, it can outcompete cesium for uptake sites, thereby reducing the amount of cesium accumulated in the organism [63]. On the other hand, the weak negative correlation suggests the absence of a direct relationship between ^40^K and ^137^Cs. While ^40^K, as a naturally occurring potassium isotope, follows tightly regulated metabolic pathways, the uptake of anthropogenic ^137^Cs is more likely influenced by site-specific environmental contamination. The lack of correlation between these radionuclides is noteworthy, as it indicates that the factors governing their bioavailability and transfer in mushrooms are distinct and operate independently. A number of studies suggest a competitive uptake mechanism between ^137^Cs and ^40^K, owing to their chemical similarity as alkali metals [64].

^40^K activity in mushrooms from different locations did not show significant differences between locations, indicating a small effect of location on ^40^K activity. This suggests that potassium uptake by mushrooms may be regulated by biological mechanisms independent of soil content, possibly influenced by species-specific absorption rates or varying soil bioavailability. Unlike ^137^Cs, which shows clear spatial dependency linked to contamination levels, ^40^K seems to follow a different uptake pattern that is less influenced by external soil concentrations.

Mushroom ^40^K activity across municipalities ranged from 1008.91 Bq/kg in Nowinka to 1400.00 Bq/kg in Narewka, while soil activity varied from 219.11 Bq/kg in Narew to 405.80 Bq/kg in Nowinka. Spearman’s rank correlation revealed a very weak positive association between soil and mushroom ^40^K levels (Rho = 0.11, *p* = 0.872, n = 6), indicating limited influence of soil ^40^K on fungal uptake.

Power analysis suggested low statistical power, implying that the non-significant result may reflect insufficient sample size rather than actual equivalence among locations. Instead, uptake appeared species-specific: *Hydnum repandum* (median: 1787.5 Bq/kg) and *Tricholoma portentosum* exhibited high ^40^K activity, while *Suillus bovinus* showed markedly lower values (median: 657.9 Bq/kg). Narrow interquartile ranges across locations (e.g., 352.10–625.47 Bq/kg) further highlight the stability of ^40^K concentrations, governed by its essential metabolic role and tight homeostatic regulation in fungi [31]. These results emphasize that species-specific physiological mechanisms, rather than soil ^40^K variability, primarily determine potassium uptake in mushrooms.

### 4.4. Reference Level

According to Council Regulation (Euratom) 2016/52 of 15 January 2016 [65], the maximum permitted activity concentration of ^137^Cs in foodstuffs intended for adult consumption, such as mushrooms, must not exceed 1250 Bq/kg. For concentrated or dried products, this level is to be calculated based on the reconstituted form of the product, as ready for consumption. In other words, the limit of 1250 Bq/kg applies not to the dried product itself, but to its edible form after typical preparation, which usually involves rehydration. In practice, dried mushrooms are rehydrated by adding water in a ratio of approximately 1:8 to 1:10, which significantly reduces the actual radionuclide concentration in the final portion consumed.

Although these regulations are formally binding across all EU Member States, differences in enforcement practices or in the interpretation of the reconstitution principle may occur, depending on national guidelines regarding dilution. This is particularly relevant for dried mushrooms, where radionuclide concentrations can increase substantially due to water loss during processing. In our study, certain species—such as *Sarcodon imbricatus*—exceeded 1900 Bq/kg ^137^Cs in dry mass. However, when recalculated based on a standard 1:9 rehydration ratio, the activity levels fell well below the 1250 Bq/kg threshold. The same applies to other commonly traded species, including *Suillus variegatus* and *Cortinarius caperatus*.

In this context, it is important to emphasize that all mushroom species analyzed in this study—including dried samples—remained compliant with the reference activity level of 1250 Bq/kg for ^137^Cs, as stipulated by Regulation (Euratom) 2016/52. Even in species with elevated ^137^Cs levels in dry form, reconstitution to the edible state resulted in values safely below the regulatory limit. These findings confirm the radiological safety of wild mushrooms from the studied region, both for domestic consumption and for export, within the current legal framework.

### 4.5. Effective Dose

Ionizing radiation is a natural component of the environment. Background radiation, primarily originating from long-lived natural sources, accounts for the majority of the annual dose received by the global population. The main contributors include radon gas emitted from the Earth’s crust, terrestrial radionuclides, and cosmic radiation, while contributions from anthropogenic sources such as nuclear accidents or historical weapons testing are comparatively minor. In Poland, the average annual effective dose from natural background radiation is estimated at approximately 2.4 mSv [66]. It should be emphasized that this regulatory limit refers to additional, controllable exposure from practices of ingestion of contaminated food, and does not include natural background radiation, which is assessed separately. In the absence of region-specific background data, a default background average of 2.4 mSv/year is commonly used to contextualize total exposure [43]. Accordingly, the effective doses calculated in this study—being orders of magnitude lower than both natural background and the regulatory threshold—indicate negligible radiological risk from mushroom consumption in the studied region, even under high consumption or dried-product scenarios.

Although ^40^K is an essential element with tightly regulated concentrations in the human body, its intake remains a measurable contributor to internal exposure and is commonly included in dose assessments for comparative purposes. This approach is consistent with international recommendations (e.g., UNSCEAR 2000; ICRP Publication 119) and aligns with national radiological protection practices in Poland. The Polish State Atomic Energy Agency (PAA) annually publishes a national report assessing the total effective dose received by the general population, incorporating both external and internal exposures. According to current legislation (Regulation of the Council of Ministers of 9 September 2021, Dz.U. 2021 poz. 1657), specific dose conversion coefficients are used for ingestion by adults, including 6.2 × 10^−9^ Sv/Bq for ^40^K and 1.3 × 10^−8^ Sv/Bq for ^137^Cs. The same coefficients were applied in the present study to ensure methodological consistency with national monitoring standards. The calculated dose from ^40^K is therefore not intended to indicate radiological risk, but to serve as a baseline for comparison with artificial radionuclides in the environment.

In this study, annual effective doses were calculated based on radionuclide activities in 1 kg of fresh mushroom species. For ^137^Cs, the dose ranged from 0.02 µSv in *Macrolepiota procera* to 2.32 µSv in *Sarcodon imbricatus*. These values are notably lower than those reported by Czech researchers (1986–2012), where annual doses from mushroom consumption ranged from 0.006 to 6 mSv [67]. In Poland, studies from the 1990s indicated that consuming 100 g of dried mushrooms could result in a dose of up to 0.22 mSv [68].

Studies conducted in Poland and Ukraine more than 30 years after the Chernobyl accident continue to report significant levels of ^137^Cs in food products originating from forested areas. Increased consumption of wild mushrooms and forest fruits contributes to higher internal exposure, with mushrooms and game meat (from wild forest animals) being the primary contributors [69].

In the present study, conducted in the north-eastern region of Poland, low levels of radiological contamination from ^137^Cs were detected in the analyzed mushroom species. It is also important to note that wild mushrooms are not typically consumed raw but rather after processing, such as blanching, frying, boiling, or pickling. According to previous research, culinary processing significantly reduces the activity concentrations of both radiocaesium and potassium in mushroom fruiting bodies [70]. The percentage reduction of ^137^Cs varies depending on the method of culinary treatment: washing reduces activity by approximately 20%, boiling by 40–97%, salting by 36–63%, frying by around 70%, and pickling by 59–73% [71]. Consequently, the activity concentrations of radionuclides in the studied mushroom species would be substantially lower following standard culinary preparation. In contrast to culinary treatments such as boiling, which reduce radionuclide content through leaching, the drying process removes water but retains the total radionuclide load. As a result, activity concentrations of radionuclides, particularly ^137^Cs and ^40^K, appear significantly higher in dried mushrooms when expressed per unit of mass (e.g., Bq/kg d.m.). This is a concentration effect rather than an increase in contamination. Consequently, dried mushrooms may represent a more significant source of internal exposure if consumed in substantial quantities without further processing.

### 4.6. Limitations

This study was specifically designed to assess internal exposure from the ingestion of wild mushrooms, focusing on ^137^Cs and ^40^K activity concentrations. Other radiological exposure pathways—such as external irradiation from soil or ambient background—were not within the scope of this research. As such, the presented dose estimates reflect ingestion-related exposure only and do not represent total environmental radiological burden.

The dose estimates presented in this study are based on median activity concentrations and average consumption assumptions. While this provides a useful baseline for interspecies comparison, it does not reflect the ICRP-defined concept of a representative person, who may experience higher exposure due to increased intake or other individual factors. Future studies could include dose modeling for representative consumers to more accurately assess radiological risk in sensitive or highly exposed subgroups.

## 5. Conclusions

This study provides updated, region-specific radiological data on wild edible mushrooms from north-eastern Poland, an area of both ecological and economic importance. Despite detectable levels of ^137^Cs—up to 159.0 Bq/kg fresh mass—none of the analyzed samples exceeded regulatory limits, including those processed by drying and evaluated according to reconstitution principles under Council Regulation (Euratom) 2016/52.

The continued detectability of ^137^Cs in wild mushrooms from industrially unpolluted areas, more than three decades after the Chernobyl accident, highlights the long-term environmental persistence of radiocaesium. The substantial interspecies variability observed suggests that physiological and ecological factors significantly influence uptake, warranting further study.

The observed interspecies variability in ^137^Cs concentrations, along with a strong positive correlation between ^137^Cs in soil and mushroom dry matter, highlights the importance of species selection and site characteristics in radiological assessments. Monitoring should prioritize species with higher uptake potential, such as *Sarcodon imbricatus*, *Hydnum repandum*, and *Tricholoma portentosum*, particularly in historically contaminated areas.

Overall, the results confirm the radiological safety of wild mushrooms from this region under current consumption scenarios and emphasize the relevance of considering both environmental exposure and post-harvest processing when evaluating food safety and trade potential.

## Figures and Tables

**Table 1:** Mushroom species, number of samples collected, and their moisture content.

No.	Species of Mushrooms	n	Water Content	
			Median	Min–Max (%)
1.	*Armillaria* sp.	29	92.83	84.59–94.05
2.	*Boletus edulis*	27	89.46	85.75–91.58
3.	*Cantharellus cibarius*	11	91.27	84.77–93.08
4.	*Cortinarius caperatus*	15	92.50	91.96–93.85
5.	*Hydnum repandum*	2	93.99	92.20–95.77
6.	*Imlaria badia*	31	92.00	74.29–94.78
7.	*Lactarius deliciosus*	2	85.45	80.96–89.95
8.	*Leccinum aurantiacum*	3	93.50	87.91–94.19
9.	*Macrolepiota procera*	31	88.42	66.48–93.05
10.	*Russula aeruginea*	4	93.20	90.12–93.44
11.	*Sarcodon imbricatus*	8	91.87	88.18–95.37
12.	*Suillus bovinus*	11	90.45	86.10–96.56
13.	*Suillus grevillei*	4	93.56	89.34–94.77
14.	*Suillus luteus*	21	92.54	85.14–96.21
15.	*Suillus variegatus*	14	91.09	89.41–93.53
16.	*Tricholoma equestre*	5	91.33	85.20–92.10
17.	*Tricholoma portentosum*	4	92.42	90.79–93.45
18.	*Xerocomellus chrysenteron*	4	91.55	88.88–93.60
19.	*Xerocomus subtomentosus*	3	91.00	90.48–91.45

**Table 2:** Activity concentration of ^40^K and ^137^Cs in collected mushrooms calculated per f.m. and d.m.

No.	Species	n	Activity in f.m.		Activity in d.m.	
			^40^K (Bq/kg)	^137^Cs (Bq/kg)	^40^K (Bq/kg)	^137^Cs (Bq/kg)
1	*Armillaria* sp.	29	109.9 (103.2; 122.9)	7.36 (2.89; 20.4)	1567.0 (1382.6; 1696.3)	95.2 (2.89; 253.6)
2	*Boletus edulis*	27	85.9 (74.5; 105.0)	40.9 (21.1; 62.2)	813.8 (725.0; 1010.8)	373.8 (150.3; 516.9)
3	*Cantharellus cibarius*	11	104.6 (75.9; 155.3)	29.2 (17.1; 36.0)	1371.3 (850.0; 1435.5)	291.2 (97.8; 372.4)
4	*Cortinarius caperatus*	15	85.6 (82.3; 95.6)	63.6 (49.9; 99.8)	1156.0 (1097.1; 1239.8)	845.9 (646.9; 1250.4)
5	*Hydnum repandum*	2	109.6 (90.1; 129.2)	35.5 (33.8; 37.1)	1787.5 (1666.5; 1908.5)	628.8 (496.4; 761.2)
6	*Imlaria badia*	31	91.7 (78.6; 102.4)	61.9 (39.8; 79.8)	1112.5 (938.2; 1141.4)	696.4 (393.9; 855.2)
7	*Lactarius deliciosus*	2	118.0 (90.0; 146.0)	34.9 (32.7; 37.2)	830.9 (765.9; 895.9)	255.0 (206.9; 303.1)
8	*Leccinum aurantiacum*	3	82.0 (67.6; 106.4)	8.92 (5.73; 9.94)	1200.6 (1039.5; 1225.1)	90.6 (43.5; 90.4)
9	*Macrolepiota procera*	31	119.0 (106.2; 179.0)	0.94 (0.56; 1.30)	1044.6 (923.3; 1147.4)	8.94 (5.46; 14.49)
10	*Russula aeruginea*	4	107.5 (98.6; 120.1)	36.5 (6.3; 72.8)	1464.6 (1387.8; 1557.6)	532.5 (112.0; 978.2)
11	*Sarcodon imbricatus*	8	89.3 (57.2; 109.4)	159.0 (135.1; 175.3)	1057.2 (933.7; 1122.1)	1976.7 (1414.7; 2267.0)
12	*Suillus bovinus*	11	64.4 (59.4; 68.8)	84.1 (55.0; 99.6)	657.9 (626.1; 708.9)	827.8 (326.5; 827.8)
13	*Suillus grevillei*	4	66.1 (64.4; 75.1)	23.2 (17.6; 29.2)	993.9 (935.8; 1088.6)	279.8 (203.8; 343.9)
14	*Suillus luteus*	22	74.9 (60.5; 98.2)	38.4 (25.8; 58.1)	1173.6 (873.5; 1243.5)	501.0 (297.0; 723.1)
15	*Suillus variegatus*	14	66.6 (58.7; 77.5)	90.2 (71.9; 107.6)	753.5 (711.9; 794.3)	1083.5 (704.1; 1150.2)
16	*Tricholoma equestre*	5	150.2 (119.2; 151.2)	30.7 (22.5; 68.1)	1500.6 (1400.4; 1731.9)	259.2 (81.0; 667.5)
17	*Tricholoma portentosum*	4	134.1 (106.3; 143.5)	45.7 (32.3; 53.6)	1724.7 (1623.2; 1716.7)	647.3 (560.7; 811.7)
18	*Xerocomellus chrysenteron*	4	126.1 (103.5; 140.9)	61.4 (32.5; 88.0)	1338.0 (1201.6; 1363.7)	718.7 (504.4; 1039.8)
19	*Xerocomus subtomentosus*	3	129.6 (129.6; 135.8)	5.02 (3.84; 9.68)	1427.6 (1361.6; 1504.4)	55.8 (31.0; 90.9)

Note: n refers to the number of samples for each species. Values are presented as Mdn (Q1; Q3), where Mdn is the median (50th percentile), Q1 is the 25th percentile, and Q3 is the 75th percentile. Data were obtained from edible mushroom samples collected in north-eastern Poland between 2017 and 2021. The activity concentrations of ^40^K and ^137^Cs were measured by gamma spectrometry in dry mass samples. Values are rounded to one or two decimal places based on the magnitude and precision of the measured activity (Bq/kg).

**Table 3:** Soil-to-mushroom transfer factors for ^40^K and ^137^Cs.

No.	Species	n	TF	
			Me Min–Max	
			^40^K	^137^Cs
1	*Armillaria* sp.	29	5.20 3.40–8.02	4.17 0.31–13.65
2	*Boletus edulis*	27	3.20 0.25–5.12	9.53 0.28–32.45
3	*Cantharellus cibarius*	11	3.44 2.17–5.31	10.17 2.13–34.46
4	*Cortinarius caperatus*	15	5.06 4.04–5.64	16.26 5.60–28.61
5	*Hydnum repandum*	2	7.99 7.45–8.53	10.70 8.45–12.96
6	*Imlaria badia*	31	3.87 2.31–5.60	14.07 4.75–55.80
7	*Lactarius deliciosus*	2	3.42 3.41–3.42	3.93 3.52–4.33
8	*Leccinum aurantiacum*	3	4.58 4.14–4.65	2.34 1.30–8.21
9	*Macrolepiota procera*	31	3.85 1.75–6.17	0.24 0.02–2.21
10	*Russula aeruginea*	4	6.03 4.81–6.23	12.37 1.60–16.81
11	*Sarcodon imbricatus*	8	3.32 2.79–5.45	41.86 29.43–61.11
12	*Suillus bovinus*	11	2.80 2.03–3.11	14.09 5.65–24.17
13	*Suillus grevillei*	4	2.75 2.33–3.09	11.33 8.62- 13.18
14	*Suillus luteus*	22	3.60 1.83–6.82	12.27 3.30–77.17
15	*Suillus variegatus*	14	3.21 1.38–3.65	18.44 3.92–24.45
16	*Tricholoma equestre*	5	5.44 4.23–5.87	5.49 1.16–26.43
17	*Tricholoma portentosum*	4	6.01 5.10–7.61	11.80 7.10–12.49
18	*Xerocomellus chrysenteron*	4	5.73 5.14–6.84	12.23 1.78–16.35
19	*Xerocomus subtomentosus*	3	4.19 4.06–5.83	2.57 1.31–10.53

Me—median.

**Table 4:** Estimated effective dose from the consumption of 1 kg and 5 kg of fresh mushrooms and 1 kg of dried mushrooms.

No.	Species	Mean Effective Doses					
		^137^Cs			^40^K		
		1 kg of Fresh Mushrooms	5 kg of Fresh Mushrooms	1 kg of Dry Mushrooms	1 kg of Fresh Mushrooms	5 kg of Fresh Mushrooms	1 kg of Dry Mushrooms
		µ Sv					
1.	*Armillaria* sp.	0.17	0.85	2.37	0.76	3.78	9.64
2.	*Boletus edulis* Bull.	0.47	2.35	4.48	0.54	2.70	5.02
3.	*Cantharellus cibarius* Fr.	0.56	2.80	5.26	0.70	3.50	7.07
4.	*Cortinarius caperatus* (Pers.) Fr.	1.02	5.1	13.58	0.56	0.53	7.58
5.	*Hydnum repandum* L.	0.46	2.30	8.17	0.55	2.75	11.08
6.	*Imleria badia* (Fr.) Fr.	0.85	4.25	9.8	0.61	3.05	6.95
7.	*Lactarius deliciosus* (L.) Pers.	0.45	2.25	3.32	0.73	3.65	5.15
8.	*Leccinum aurantiacum* (Bull.) Gray	0.10	0.5	1.18	0.61	3.05	7.54
9.	*Macrolepiota procera* (Scop.) Singer	0.02	0.1	0.16	0.88	4.40	6.74
10.	*Russula aeruginea* Lindblad ex Fr.	0.55	2.75	6.82	0.68	3.40	9.23
11.	*Sarcodon imbricatus* (L.) P. Karst.	2.32	11.6	29.30	0.55	2.75	6.59
12.	*Suillus bovinus* (L.) Roussel	0.98	4.9	10.17	0.40	2.00	4.27
13.	*Suillus grevillei* (Klotzsch) Singer	0.31	1.55	4.35	0.46	2.30	6.48
14.	*Suillus luteus* (L.) Roussel	0.54	2.70	6.86	0.52	2.60	6.95
15.	*Suillus variegatus* (Sw.) Richon & Roze	1.11	5.55	12.70	0.39	1.95	4.68
16.	*Tricholoma equestre* (L.) P. Kumm.	0.73	3.65	8.53	0.93	4.65	9.29
17.	*Tricholoma portentosum* (Fr.) Quel.	0.60	3.00	7.96	0.82	4.10	10.61
18.	*Xerocomellus chrysenteron* (Bull.) Šutara	0.77	3.85	8.130	0.73	3.65	8.48
19.	*Xerocomus subtomentosus* (L.) Quél.	0.10	0.50	1.03	0.82	4.10	9.17

## Data Availability

Data available from the authors upon request.

## Abbreviations

The following abbreviations are used in this manuscript:

d.m.	dry mass
f.m.	fresh mass
HPGe	High-purity germanium
ICRP	International Commission on Radiological Protection
MANOVA	multivariate analysis of variance
PCA	principal component analysis
Rho	rank correlation coefficient

## Appendix A

**Table A1:** Number of samples and locations.

No.	Species	Type of Nutritional Strategy	Location	Number of Samples n = 230
1.	*Armillaria* sp.	saprotrophic	Hajnówka	2
			Narew	4
			Supraśl	19
			Michałowo	4
2.	*Boletus edulis*	mycorrhizal	Hajnówka	6
			Narew	10
			Nowinka	8
			Supraśl	3
3.	*Cantharellus cibarius*	mycorrhizal	Hajnówka	2
			Narew	2
			Nowinka	3
			Michałowo	1
			Supraśl	3
4.	*Cortinarius caperatus*	mycorrhizal	Hajnówka	5
			Narew	8
			Supraśl	2
5.	*Hydnum repandum*	mycorrhizal	Narew	2
6.	*Imlaria badia*	mycorrhizal	Hajnówka	5
			Narew	11
			Nowinka	4
			Michałowo	1
			Supraśl	10
7.	*Lactarius deliciosus*	mycorrhizal	Hajnówka	1
			Narew	1
8.	*Leccinum aurantiacum*	mycorrhizal	Hajnówka	1
			Narew	1
			Narewka	1
9.	*Macrolepiota procera*	saprotrophic	Hajnówka	1
			Narew	8
			Narewka	2
			Nowinka	3
			Michałowo	3
			Supraśl	14
10.	*Russula aeruginea*	saprotrophic	Hajnówka	1
			Narew	2
			Narewka	1
11.	*Sarcodon imbricatus*	mycorrhizal	Narew	2
			Supraśl	6
12.	*Suillus bovinus*	mycorrhizal	Hajnówka	1
			Narew	7
			Nowinka	1
			Supraśl	2
13.	*Suillus grevillei*	mycorrhizal	Nowinka	3
			Supraśl	1
14.	*Suillus luteus*	mycorrhizal	Hajnówka	3
			Narew	6
			Michałowo	2
			Supraśl	9
			Narewka	1
15.	*Suillus variegatus*	mycorrhizal	Hajnówka	2
			Narew	10
			Supraśl	2
16.	*Tricholoma equestre*	mycorrhizal	Hajnówka	2
			Narew	1
			Supraśl	2
17.	*Tricholoma portentosum*	mycorrhizal	Hajnówka	1
			Narew	1
			Supraśl	2
18.	*Xerocomellus chrysenteron*	mycorrhizal	Narew	4
19.	*Xerocomus subtomentosus*	mycorrhizal	Narew	1
			Narewka	1
			Nowinka	1
